# Quantitative volumetric Raman imaging of three dimensional cell cultures

**DOI:** 10.1038/ncomms14843

**Published:** 2017-03-22

**Authors:** Charalambos Kallepitis, Mads S. Bergholt, Manuel M. Mazo, Vincent Leonardo, Stacey C. Skaalure, Stephanie A. Maynard, Molly M. Stevens

**Affiliations:** 1 Department of Materials, Imperial College London, London SW7 2AZ, UK; 2 Department of Bioengineering, Imperial College London, London SW7 2AZ, UK; 3 Institute of Biomedical Engineering, Imperial College London, London SW7 2AZ, UK

## Abstract

The ability to simultaneously image multiple biomolecules in biologically relevant three-dimensional (3D) cell culture environments would contribute greatly to the understanding of complex cellular mechanisms and cell–material interactions. Here, we present a computational framework for label-free quantitative volumetric Raman imaging (qVRI). We apply qVRI to a selection of biological systems: human pluripotent stem cells with their cardiac derivatives, monocytes and monocyte-derived macrophages in conventional cell culture systems and mesenchymal stem cells inside biomimetic hydrogels that supplied a 3D cell culture environment. We demonstrate visualization and quantification of fine details in cell shape, cytoplasm, nucleus, lipid bodies and cytoskeletal structures in 3D with unprecedented biomolecular specificity for vibrational microspectroscopy.

## Abstract (web summary)

Advances in chemical imaging capability can add to our understanding of complex cellular systems. Here the authors develop a framework for label-free quantitative volumetric Raman spectroscopic imaging and use it to visualize and quantify biomolecules in various 3D cellular systems.

Cell culture systems that better recapitulate the physiological conditions and environment a cell experiences *in vivo* can improve our understanding of cellular behaviour. The development of three-dimensional (3D) cell culture systems has been effectively applied in several fields including developmental biology, tissue engineering and drug discovery^1^. Indeed, a plethora of new 3D culture systems are continuously being developed to aid this transition from two-dimensional (2D) systems in life sciences^2^,^3^. These culture systems heavily rely on optical imaging modalities to provide biological information. Currently, 3D culture systems are studied using optical microscopy techniques, with confocal fluorescence microscopy being the gold standard^4^. However, conventional confocal microscopy is semi-quantitative and requires labelling that may severely influence cell function and intracellular processes^5^. Hence, there is a great and unmet need to introduce endogenous techniques that can study cells in highly relevant 3D environments while providing quantitative biomolecular information of multiple components simultaneously and non-destructively.

Several optical imaging techniques based on endogenous biomolecules have previously been used to provide detailed visualization of the morphology and spatial distribution of biological structures^4^,^6^,^7^,^8^,^9^,^10^,^11^,^12^. One of these, Raman spectroscopy, which is an inelastic light scattering technique, can provide label-free biochemical information. Raman spectroscopy-based imaging studies on cellular systems have been mostly applied using non-confocal settings associated with challenges arising from substrate background signals and poor *z*-axis resolution giving rise to overlapping spectral signatures. This naturally compromises the biomolecular specificity achievable using multivariate analysis. The development of confocal Raman spectroscopy has enabled greater depth resolution^13^; however, the vast majority of cellular confocal Raman spectroscopy studies have still been limited to imaging of a single layer, which might compromise biomolecular quantification. Of the few early reports of 3D confocal Raman imaging^14^,^15^, these are not yet coupled to biomolecular quantification.

Here we introduce a comprehensive computational framework, namely quantitative volumetric Raman imaging (qVRI) to visualize, identify and quantify biomolecules in 2D and 3D cell culture systems. We use qVRI to image: human pluripotent stem cells and monocytes/macrophages in conventional culture systems, as well as mesenchymal stem cells in 3D biomaterials. Our results reveal an intimate relationship between spatial resolution and biomolecular specificity achievable using spectral unmixing techniques. By taking advantage of this, we demonstrate the visualization of fine details in 3D cell shape, cytoplasm, nucleus, protein rich clusters, intracellular lipid bodies, membrane lipids and submicron-sized cytoskeletal structures with unprecedented biomolecular specificity for vibrational microspectroscopy. Further, we demonstrate for the first time a volumetric quantification of endogenous biomolecules using 3D Raman imaging datasets that allows us to spatially monitor complex biological processes like differentiation within a 3D cell culture system.

## Results

### qVRI computational framework

**Figure 1:**
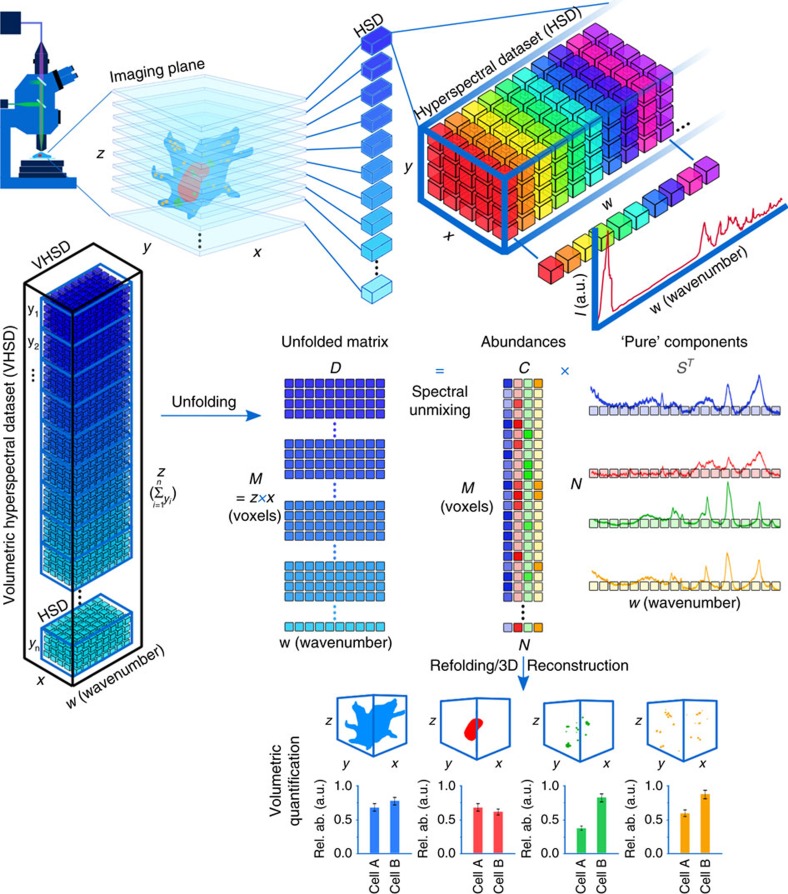
Schematic illustration of qVRI imaging process, from data collection and spectral unmixing to 3D reconstruction and quantification. The confocal microscope provides control over *x × y × z* dimensions of the sample position for 3D imaging. Each imaging plane is described by a hyperspectral dataset. Hyperspectral datasets are 3D datasets with *x × y* (number of pixels in a single imaging plane) spatial dimensions and *w* (wavenumbers) spectral dimension. Each voxel in 3D is associated with a single Raman spectrum. Combining hyperspectral datasets from multiple imaging planes creates a volumetric hyperspectral dataset with *z × x* spatial dimensions and *z* being equal to the sum of *y*_*n*_ imaging planes. For spectral unmixing analysis the volumetric hyperspectral dataset is unfolded to form a matrix *D=M × w* with *M=z × x*. *D* is unmixed using N number of ‘pure' components (e.g., here *N*=4) into two matrices *C* and *S^T^. C* contains the relative abundance values of the pure components in each voxel in an *M × N* matrix with every column associated to one component. *S^T^* is an *N × w* matrix containing a ‘pure' component spectrum in every row. Each column of *C* contains all the spatial information needed to reconstruct every components' 3D architecture by refolding it to the original *x × y × z* dimensions. Each voxel contains the concentration profile of the reconstructed component. The number of voxels within an isosurface at a chosen threshold can be used as a metric for quantification, comparing experimental conditions (e.g., comparing Cell A to Cell B).

Our developed computational framework is tailored to handle volumetric hyperspectral datasets measured from a *z*-stack of Raman images (Fig. 1). First, all hyperspectral datasets collected from each *z* layer are assembled and unfolded into a single data matrix. Each Raman spectrum of a hyperspectral dataset is then pre-processed individually (see Methods). Next, the unfolded volumetric hyperspectral dataset can be either directly reconstructed based on univariate peak intensities or fed to a spectral unmixing algorithm. Here we applied vertex component analysis (VCA) due to its computational efficiency over other algorithms^16^. Briefly, VCA assumes that the cell volume contains a number of voxels with nearly pure components known as ‘endmembers'. Endmembers are real spectra obtained directly from the pre-processed volumetric hyperspectral dataset and are assumed to represent the voxels in the dataset containing the purest amounts of specific biomolecules. For high resolution confocal Raman imaging, these endmembers essentially represent the biomolecular architecture of the entire cell and its organelles. Consequently, biomolecular relative abundance values can be associated with each voxel^17^. Finally, each biomolecular component can be refolded for 3D volumetric visualization and quantification.

### 3D visualization of pluripotent stem cells and cardiomyocytes

**Figure 2:**
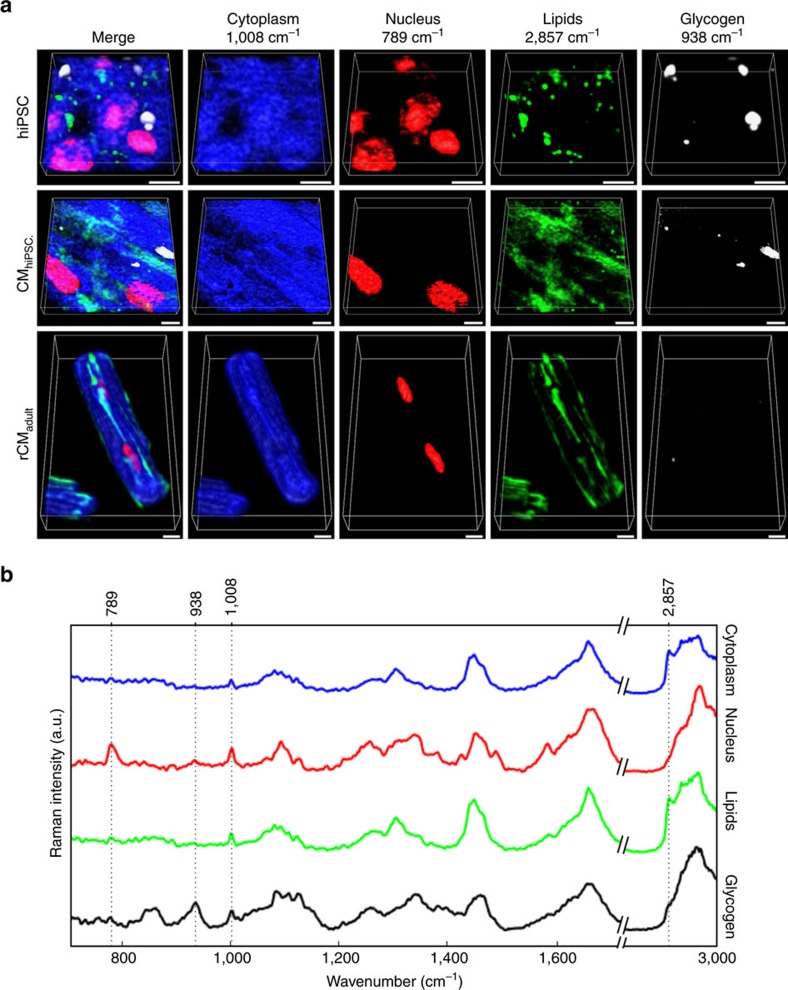
3D visualization of representative pluripotent stem cells and CMs. (**a**) 3D volumes of intensity distributions of hiPSCs (*n*=4 colonies), hiPSC-derived CMs (*n*=4 monolayers) and adult rat ventricular CMs (*n*=2 cells) for selected bands: 1,008 cm^−1^ for cell cytoplasm (blue), 789 cm^−1^ for cell nucleus (red), 2,857 cm^−1^ for lipids (green), 485 cm^−1^ for glycogen (white) and a merge of all components. (**b**) Representative Raman spectra of each subcellular component (cytoplasm (blue), nucleus (red), lipids (green), glycogen (black)), indicating the band used to reconstruct the 3D intensity volumes. Scale bar, 10 μm.

To demonstrate the developed approach we imaged human induced pluripotent stem cells (hiPSCs), hiPSC-derived cardiomyocytes (CMs) and adult rat ventricular CMs since they present a well-known and defined 3D morphology and distinct biochemical composition^18^,^19^,^20^,^21^,^22^. Needing to accurately determine the degree of maturation in hiPSC-CMs after *in vitro* differentiation is one of the main constraints for their bioapplication^23^. As a consequence, a comprehensive characterization of each maturation step is highly valuable for both the progress and translation of this technology^24^. In the work reported here, all volumetric Raman imaging was performed using a 50 μm pinhole. We first performed univariate imaging of distinct vibrational modes. The intensities of specific Raman peaks were volumetrically reconstructed highlighting the cell's main biochemical components and visualizing their 3D morphology (Fig. 2). We used the highly specific molecular marker of phenylalanine (1,008 cm^−1^) to visualize protein content^25^. DNA was visualized using the O–P–O band (789 cm^−1^) while the CH_2_ symmetric stretching highlighted areas rich in lipid structures (2,857 cm^−1^). The 3D reconstruction of hiPSCs emulates the dense morphology of human pluripotent stem cell colonies^26^. hiPSC-derived CMs were ∼3 μm in height, which matches previous reports of their particularly flat nature^27^. Adult ventricular CM reconstruction very clearly showed a binucleated mature cell with the expected elongated rod-like form and sarcomeric protein strips^24^. hiPSC-derived CMs also exhibited early stages of protein alignment, but not as distinct as adult CMs. Glycogen, an energy-storing polysaccharide, showed a differential presence, with distinct peaks associated with glycogen in the hiPSC colonies and hiPSC-derived CMs, that are not seen in adult CMs^23^. Therefore, the presence of glycogen can be related to the degree of maturation of hiPSC-CM, highlighting the ability of our novel approach to provide relevant data on the quality of hiPSC-derived CMs. To our knowledge, this is the first label-free technique able to provide 3D information in this regard^23^.

### Volumetric quantification of monocytes and macrophages

**Figure 3:**
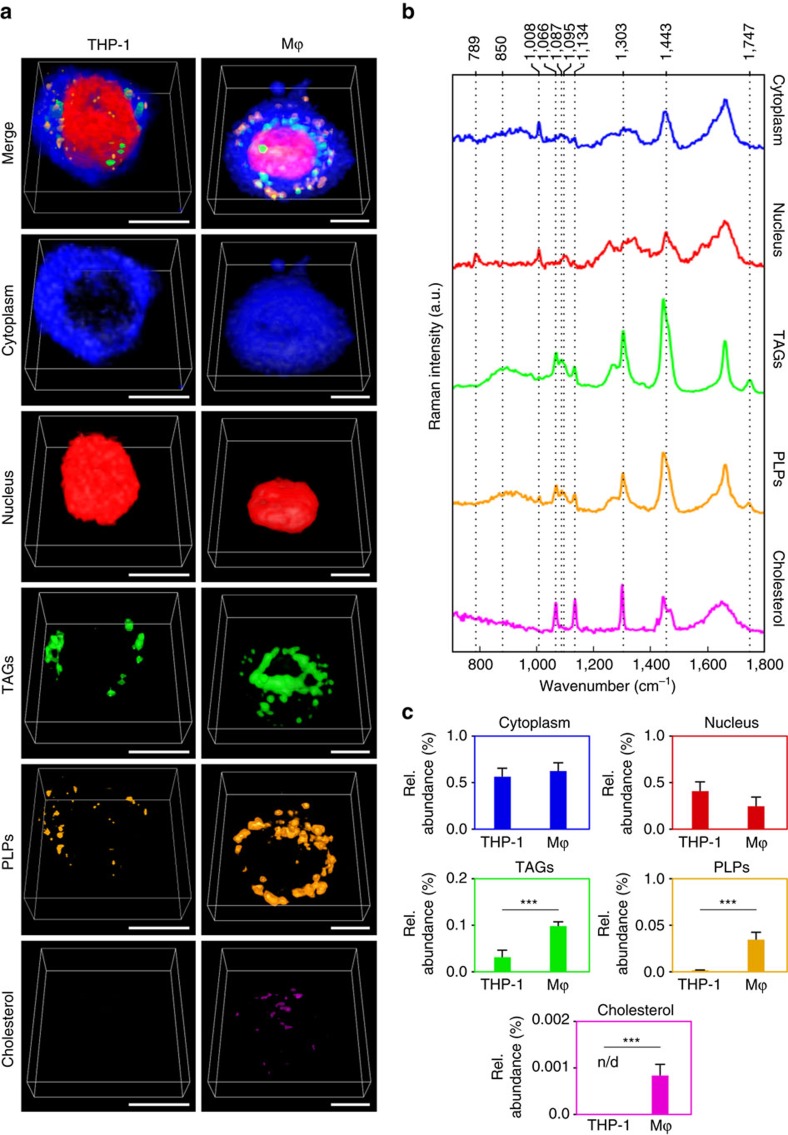
Lipid analysis in monocyte to macrophage differentiation. (**a**) qVRI identifies the main subcellular components of THP-1 cells (*n*=4 cells) and THP-1 differentiated macrophages (Mϕ) (*n*=4 cells) representative cells shown, and their corresponding (**b**) endmember Raman spectra from VCA (showing five components); from top to bottom cytoplasm (blue), nucleus (red), triacylglycerols (green), phospholipids (orange), cholesterol (magenta). (**c**) Bar chart of mean abundance values for each subcellular component showing significant differences between the two cells for TAG (two sample *t*-test, *P*<0.001), PLP (two sample *t*-test, *P*<0.001) and cholesterol (two sample *t*-test, *P*<0.001), ***P<0.001 (n/d, non-detectable). Error bars represent one standard deviation around the mean. Scale bar, 10 μm.

We then aimed to demonstrate the volumetric quantification ability of qVRI. We followed the differentiation of monocytes (THP-1 cells) into macrophages (Mϕ) and characterized their complex lipid composition. A heterogeneous population of lipids is known to have numerous roles in phagosome formation and maturation in Mϕ (ref. 28). Therefore, understanding the role of each lipid subtype involved in their endocytic pathway provides crucial information for the immune responsiveness of these cells. To improve the molecular specificity compared to univariate imaging, the volumetric hyperspectral data collected were analysed using VCA. From the analysis we identified three different types of lipids: triglycerides (TAGs), phospholipids (PLPs) and cholesterol esters (Fig. 3)^29^. We reconstructed each of the biochemical components identified (Fig. 3a). TAGs were present in dense lipid bodies in the cytoplasm. We identified PLPs located at the cell membrane as well as very small cholesterol rich regions possibly localized inside endosomes. The extracted cytoplasm spectra from the VCA show the typical phenylalanine and amide bands expected from cellular structures. The three lipid types all show strong CH_2_ twists (1,300 or 1,303 cm^−1^) and blue shifted CH_2_ deformation (1,443 cm^−1^) compared to protein rich regions, like the cytoplasm and nucleus. TAG spectra show the characteristic C=O stretching (1,747 cm^−1^) (not present in fatty acids) and some low intensity vibrations around 850–890 cm^−1^ (not present in cholesterol ester and membrane lipids), which could be related to C–O–O or CH_3_ rocking vibrations. Moreover, PLP spectra show a chain C–C stretch relating them to membrane lipids^29^. Cholesterol spectra were of very high intensity showing the specific bands expected from cholesteryl stearate with the additional shoulder at 1,443 cm^−1^ and the 1,066 cm^−1^ and 1,134 cm^−1^ bands (Fig. 3b). These results show that by using quantitative volumetric Raman spectroscopy different lipid subtypes can be identified. To compare biochemical composition of the undifferentiated THP-1 cells to the differentiated Mϕ we applied the volumetric quantification protocol (Fig. 1) by using the abundance values of the respective VCA components (see Methods). We measured similar amounts of cytoplasmic proteins and DNA (Fig. 3c), yet Mϕ showed significantly higher amounts of PLPs (two sample *t*-test, *P*<0.001). Interestingly there was an increase in TAGs compared to the THP-1 cells (two sample *t*-test, *P*<0.001). Cholesterol signals were only present in Mϕ (two sample *t*-test, *P*<0.001) (Fig. 3c). Hence, localization and characterization of lipid composition within the cytoplasm and quantification of TAG and other lipid accumulations can provide valuable insight into Mϕ-to-foam cell transition, which could help in the study of diseases like atherosclerosis^30^.

The computational framework developed maximizes the imaging capability of confocal Raman spectroscopy and enables visualization of small biomolecular accumulations. Resolving the *z* dimension improves molecular specificity achievable using spectral unmixing. To exemplify this, we simulated the Raman signal *z*-overlapping that follows standard non-confocal analysis by summing over the whole *z* dimension. We then applied the same VCA analysis and volumetric reconstruction (Supplementary Fig. 1). These results show that subcellular structures significantly overlap hindering the spectral unmixing capacity to resolve individual biomolecules such as different lipid subtypes. For instance spectral unmixing could not resolve PLPs or cholesterol from the TAG signals, and no significant differences between lipids were found following volumetric quantification (two sample *t*-test, *P*>0.05). Overall, this shows that the superior molecular specificity provided by high-resolution qVRI enables biomolecular subtyping not achievable using standard Raman techniques.

### Volumetric quantification in a 3D cell culture system

**Figure 4:**
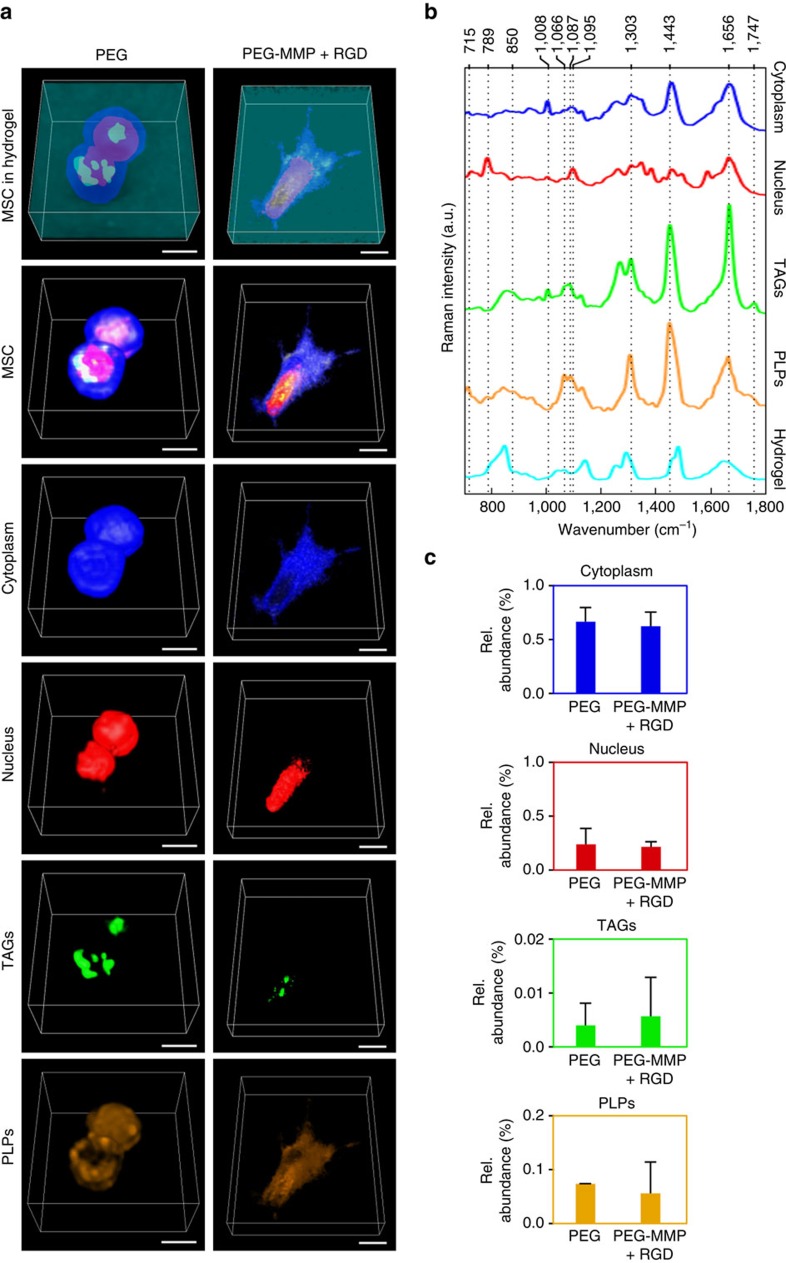
Volumetric quantification of cellular biochemical components in a 3D culture system. Representative 3D reconstructions by qVRI, of the main subcellular components of hMSCs (*n*=2 cells per hydrogel) in (**a**) a bioactive (PEG-MMP+RGD) and a bioinert (PEG) hydrogel, and their corresponding (**b**) endmember Raman spectra from VCA (showing five components); from top to bottom cytoplasm (blue), nucleus (red), triacylglycerols (green), phospholipids (orange) and hydrogel (cyan). (**c**) Bar chart of mean abundance values for each subcellular component. Error bars represent one standard deviation around the mean. Scale bar, 10 μm.

Given recent efforts in developing advanced 3D hydrogel culture systems^31^, we aimed to apply qVRI for the characterization of cellular biochemistry and morphology within a hydrogel biomaterial. We imaged hMSCs in two different 3D hydrogel culture systems, where one is bioinert in that the network is only comprised of polyethylene glycol (PEG), and the other is bioactive by incorporating biomimetic peptides for both cellular adhesion (arginylglycylaspartic acid (RGD) peptide) and degradation (matrix metalloproteinase (MMP) degradable peptide) within the hydrogels' crosslinked network (Fig. 4a). As shown here before, qVRI provides a spectral signature for the subcellular components identified (Fig. 4b). Using the intensity of the 847 cm^−1^ band (C–O–C or C–C stretching) that corresponds to the PEG network, the hydrogel can be visualized and precisely distinguished from the cells. In this culture system, the reconstruction of PLPs enabled an unprecedented visualization of the entire cell membrane. To our knowledge, this is the first time a distinct lipid signal can be resolved from a thin cell membrane that can be attributed to the high molecular specificity provided by the spectral unmixing framework. Cells within the bioinert PEG hydrogel appeared to be ‘entrapped' in the hydrogel, retaining a spherical shape, indicating little or no interaction with the material as predicted for this hydrogel. On the contrary, in the bioactive peptide-functionalized hydrogel, qVRI reconstruction reveals a more elongated cellular morphology in 3D, with filopodia extending in all directions that indicate cellular adhesion and interaction with the hydrogel network structure (see Supplementary Movie). Our biochemical volumetric quantification found no significant differences in the abundances of biochemical components comprising the cells in the two different hydrogels (two sample *t*-test, *P*>0.05), which is not surprising considering this analysis was performed after only 7 days in culture and in the absence of any differentiation factors (Fig. 4c). However, our validation within short-term culture provides proof-of-concept for the qVRI technique, which could ultimately provide highly valuable insight into biomolecular changes during the long-term culture of stem cells undergoing differentiation within different 3D culture systems, in order to elucidate with greater detail how cell–material interactions affect biological processes. Additionally, further importance lays in the label-free, non-destructive molecular specificity of qVRI, since standard methods^4^ used to characterize cells in biomaterials require labelling and often destructive sectioning in order to apply staining molecules and conduct accurate imaging. Thus, qVRI allows us to image cells non-invasively and quantify important biomolecules in a more relevant 3D culture environment. To the best of our knowledge, this is the first report of a label-free 3D reconstruction, characterization and quantification of a cell within a biomaterial.

In conclusion, our developed quantitative Raman imaging approach represents a novel label-free method for visualization of 3D cell morphology and volumetric quantification of biomolecular structures with submicron-size detail. We show that spatial resolution and molecular specificity are intimately interlinked and through spectral unmixing, we can characterize relevant biomolecules and resolve them into subtypes. We envision the developed approach being a highly valuable complementary information source for a variety of cell biology and tissue engineering applications. Finally, qVRI will open up new avenues for studying the complexities of cell–material interactions within a plethora of 3D culture systems, revealing new information about cell behaviour and function in advanced biomaterials that has been until now difficult or impossible to measure.

## Methods

### 3D confocal Raman imaging

3D Raman imaging was performed on a confocal Raman micro-spectroscope (alpha300R+, WITec, Ulm, Germany). The light source used was a 532 nm laser with the application of a × 63/1.0 NA water immersion microscope objective lens (W Plan-Apochromat, Zeiss, Oberkochen, Germany). The scattered light was directed to the spectrometer via a 50 μm fibre also acting as a pinhole providing confocality. A 600 lines per mm grating spectrograph (UHTS 300, WITec, Ulm, Germany) was used and spectra were acquired using a thermoelectrically cooled back-illuminated CCD camera (Newton DU970N-BV-353, Andor, Belfast, UK) with spectral resolution of ∼10 cm^−1^ (defined at full width at half maximum of mercury argon emission lines) and 40 mW laser power at the sample. Cells were imaged by collecting Raman images from 10 layers of 1 μm increment in the *z* direction spanning the cell volume (Supplementary Fig. 2). A 650 nm step size was used in the *x* and *y* direction for each Raman image with 0.3 s integration time and a spectral range from 0 to 3,000 cm^−1^.

### Multivariate data analysis

Each volumetric hyperspectral dataset was analysed using in-house written methods through Matlab software (2016, MathWorks). Each Raman image was first pre-processed separately and has undergone baseline correction, cosmic rays removal and smoothing using the PLS toolbox by Eigenvector Research, Inc. as shown in Supplementary Fig. 3. For the cosmic ray removal, we used a principal component analysis algorithm and outlier spectra identified by Q residuals and Hotelling's T^2^ plots were removed manually after being confirmed that they contained cosmic rays. For the spectral smoothing, we used a second order Savitzky–Golay algorithm with a 3-point window^32^. For the baseline correction, we used the weighted least squares processing method with a third order polynomial^32^ in the spectral range (700–1,800 cm^−1^). Following this, all hyperspectral images were assembled into one volumetric hyperspectral dataset and unfolded into a single matrix. The volumetric hyperspectral dataset was normalized to remove any instrument effects and make the samples comparable. VCA^16^ was used to ‘unmix' the Raman spectra and identify subcellular components within the volumetric hyperspectral datasets using the entire dataset including all cells imaged. For each application, the VCA analysis was performed using a number of components that maximized the number of spectra that could be related to a specific biological component through peak assignment and correlation to previously published work^29^. From the VCA analysis the ‘pure' spectra of each subcellular component were obtained. Following this, a non-negative least squares algorithm was used for each of the VCA obtained spectra to provide abundance values from 0 to 1 associated to each voxel in the volumetric hyperspectral dataset. By refolding each component's abundance values matrix back to its original shape, a volume describing the components' 3D architecture was plotted using Icy software (Version 1.7.3.0, BioImage Analysis unit Institut Pasteur). We then selected a threshold value, which removed the background signal. We found that values close to the average abundance value worked well for our specific application. Threshold values were fixed for each experiment and an isosurface was plotted for each component. The number of voxels within each isosurface were counted and the percentage to the total number of voxels for that component was calculated. These values were used for the volumetric quantification and comparison between different cell types^17^. Data were checked with two sample variance tests to be normally distributed. Comparisons between groups were therefore performed using an unpaired two-sided Student's *t*-test, and the result considered statistically significant *P<0.05, **P<0.01 and ***P<0.001.

### Cardiac differentiation of human pluripotent stem cells

All products are from Life Technologies (UK) unless otherwise stated. Human-induced pluripotent stem cell-derived CMs were obtained as follows. Gibco Episomal hiPSC Line was induced to differentiate by biphasic modulation of Wnt signalling^33^. Briefly, cells were routinely grown on Matrigel coated plates in Essential 8 medium. To start differentiation, cells were detached with 0.5 mM EDTA and plated at 150,000 cells cm^−2^ in Essential 8 medium supplemented with Thiazovivin (Stratech Scientific, UK). Upon confluence (approximately 24 h later), cells were exposed for 24 h to 10 μM Wnt-activatorCHIR99021 in RMPI plus 1 × B27 minus insulin. Subsequently, cultures were maintained for 48 h in RPMI plus 1 × B27 minus insulin, then for 48 h in 5 μM Wnt-inhibitor IWP4 (Tebu-bio, UK) in RPMI plus 1 × B27 minus insulin. Finally, cells were kept for 48 h in RPMI plus 1 × B27 minus insulin before being transitioned to RPMI plus 1 × B27 (complete). Beating clusters started to emerge around 9 days after the start of the differentiation. Thereafter, a metabolic selection method by depletion of glucose and supplementation with 4 mM Lactate (Sigma-Aldrich, UK)^34^ was applied for 3 days. Cells were then returned to normal RPMI plus 1 × B27 for 2 days before a second round of purification. Finally, cells were detached using TrypLE Express and plated on glass bottom chamber slides for subsequent analysis. Slides were then fixed with 4% v/v PFA and washed gently with PBS before imaging.

### Isolation of rat adult ventricular cardiomyocytes

All work was carried out under the Animals (Scientific Procedures) Act 1986 and the EU Directive 2010/63/EU. Sprague-Dawley rats were killed by cervical dislocation after anaesthesia with 3% isoflurane. Following aortic cannulation to the Langendorff setup, the hearts were perfused with normal Tyrode's solution for 5 min (in mM: 120 NaCl, 5.4 KCl, 5 MgSO_4_, 5 sodium pyruvate, 20 glucose, 20 taurine, 10 HEPES (free acid), 5 nitrilotriacetic acid, and 0.04 CaCl_2_, all from Sigma-Aldrich; pH 6.96), and finally for 9 min with a solution containing collagenase (1 mg ml^−1^; Worthington, UK) and hyaluronidase (0.6 mg ml^−1^; Sigma-Aldrich) dissolved in buffer solution (in mM: 120 NaCl, 5.4 KCl, 5 MgSO_4_, 5 sodium pyruvate, 20 glucose, 20 taurine, 10 HEPES (free acid), and 0.2 CaCl_2_; pH 7.4). The left ventricle was then removed, cut into small pieces, resuspended in collagenase/hyaluronidase solution, and shaken in a water bath at 37 °C for 5 min twice. Then the cells were filtered through a 200-μm nylon mesh and centrifuged at 500 rpm for 1 min. The cells used for experiments were re-suspended and stored in the buffer solution at room temperature. Myocytes were kept in normal Tyrode's solution until used when they were transferred to culture medium. Myocytes were plated in culture in maintenance medium (M199 (Earls' salt), 2% w/v BSA, 5 mM creatine, 5 mM taurine, 0.1 mM ascorbic acid, 2 mM carnitine, 100 U ml^−1^ penicillin, and 100 mmol l^−1^ streptomycin). The medium was changed after 2 h to remove unattached cells. Myocytes were fixed and analysed within 24 h of isolation.

### Monocyte differentiation and cell attachment to slides

THP-1 cells (CD34±) were cultured in RPMI 1640 medium with 2 mM L-glutamine, 10% FBS. THP1-derived macrophages (CD14±) were obtained by supplying 100 ng ml^−1^ of phorbol-12-myristate-13-acetate (PMA) to the THP-1 cell media. After 48 h cells differentiated from suspended monocytes to machrophages attached to the substrate. Glass or MgF_2_ slides were used as substrates. For the THP-1 cells, slides were pre-treated with BD Cell-Tak Cell and Tissue Adhesive in a 24-well tissue culture plate (BD Biosciences, 5.2 μl in 300 μl of sodium bicarbonate buffer, pH=8 for 40 min at room temperature). Following two washing steps with ddH_2_O, MgF_2_ slides were incubated with the cell suspension in PBS for 30 min. Slides were then fixed with 4% v/v PFA and washed gently with PBS before imaging.

### hMSC 3D culture and hydrogel synthesis

A 10% w/w macromer solution of eight-arm PEG-norbornene^35^,^36^ was mixed with appropriate crosslinker and adhesive peptide where indicated in αMEM supplemented with 1% v/v antibiotic/Antimycotic (A/A; Invitrogen, UK). For the bioinert hydrogel, PEG-dithiol (MW 1,000, Sigma) was used as the crosslinker at a thiol:norbornene molar ratio of 0.8:1. For the degradable hydrogel, the MMP-degradable peptide CDDGPQGIWGQC was used as the crosslinker at a thiol:norbornene molar ratio of 0.8:1, and the cell-adhesive peptide CGRGDS was added at 2.5 mM in the macromer solution. Peptides were synthesized by standard Fmoc solid-phase peptide synthesis, followed by HPLC purification (water/acetonitrile gradient with 0.1% v/v TFA) and pure peptide molecular weight verification using LCMS. The hMSCs were cultured under standard cell culture conditions (37 °C, humidified atmosphere with 5% CO^2^). Bone marrow-derived hMSC were purchased from Lonza (UK) and expanded to passage 4. The Lonza donor used for all experiments was a 29-year-old Caucasian female (#372262). The hMSCs were cultured in T175 flasks at a cell density of approximately 2,857 cells cm^−2^ in Alpha Minimal Essential Medium Glutamax-1 (αMEM; Life Technologies, UK), supplemented with 10% v/v mesenchymal stem cell grade fetal bovine serum (MSC-FBS; Life Technologies, UK) and 1% v/v A/A, changing the media every 2–3 days. Cells were encapsulated in hydrogels after reaching passage 5, following trypsinization of passage 4 cells at confluence. The hMSCs were mixed with macromer solution at 1 million cells ml^−1^ and photopolymerized with 0.05% w/w Irgacure 2959 (Sigma) into thin discs (6 mm diameter × 0.5 mm height) for 7 min with 365 nm light (5 mW cm^−2^). Hydrogels were cultured in αMEM supplemented with 10% v/v MSC FBS and 1% v/v A/A for 7 days. The hydrogels were then washed three times in Dulbecco's Phosphate Buffered saline (DPBS; Invitrogen, UK) before fixing in 4% v/v paraformaldehyde (PFA) in DPBS. The hydrogels were kept in DPBS at 4 °C until imaging.

All cell lines mentioned have tested negative for mycoplasma contamination. Checks were performed monthly using MycoAlert detection kit (Lonza, UK).

### Data availability

The raw research data supporting this paper are available at https://doi.org/10.5281/zenodo.256329.

## Additional information

**How to cite this article:** Kallepitis, C. *et al*. Quantitative volumetric Raman imaging of three dimensional cell cultures. *Nat. Commun.* **8,** 14843 doi: 10.1038/ncomms14843 (2017).

**Publisher's note**: Springer Nature remains neutral with regard to jurisdictional claims in published maps and institutional affiliations.

## Supplementary Material

Supplementary Information Supplementary Figures

Supplementary Movie 1 3D reconstruction of a hydrogel-based cell culture system. Subcellular components (cytoplasm shown in blue, nucleus in red, triglycerides in green and phospholipids in orange) of a mesenchymal stem cell expanding within a MMP-degradable PEG hydrogel, shown in cyan.
