# Supplementary material for: Quantitative volumetric Raman imaging of three dimensional cell cultures
Source: Nat Commun. 2017 Mar 22;8:14843. doi: 10.1038/ncomms14843 (PMC5364421; doi:10.1038/ncomms14843)
Supplement: Supplementary Information — Supplementary Figures [file ncomms14843-s1.pdf]

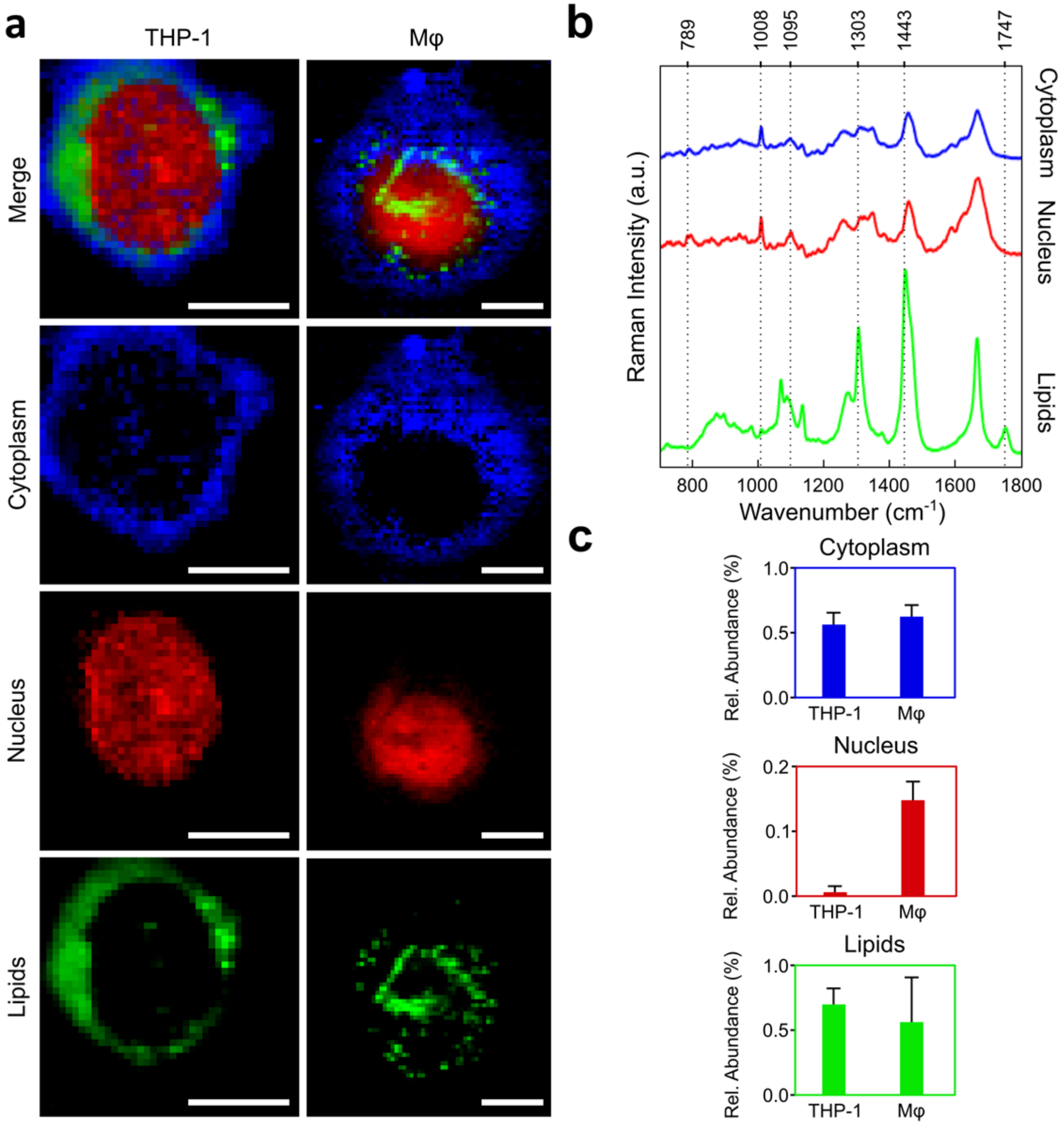

Supplementary Figure 1│ Raman spectral imaging of representative THP-1 and Mφ cells. **(a)** Identifying the main subcellular components of THP-1 cells and THP-1 differentiated macrophages (Mφ), and their corresponding **(b)** endmember Raman spectra from VCA (showing 3 components) using a z

stack projection by summing hyperspectral datasets from all imaging layers; from top to bottom cytoplasm (blue), nucleus (red), general lipids (green). **(c)** Bar chart of mean abundance values for each subcellular component. Error bars represent one standard deviation around the mean. Scale bar 10 μm.

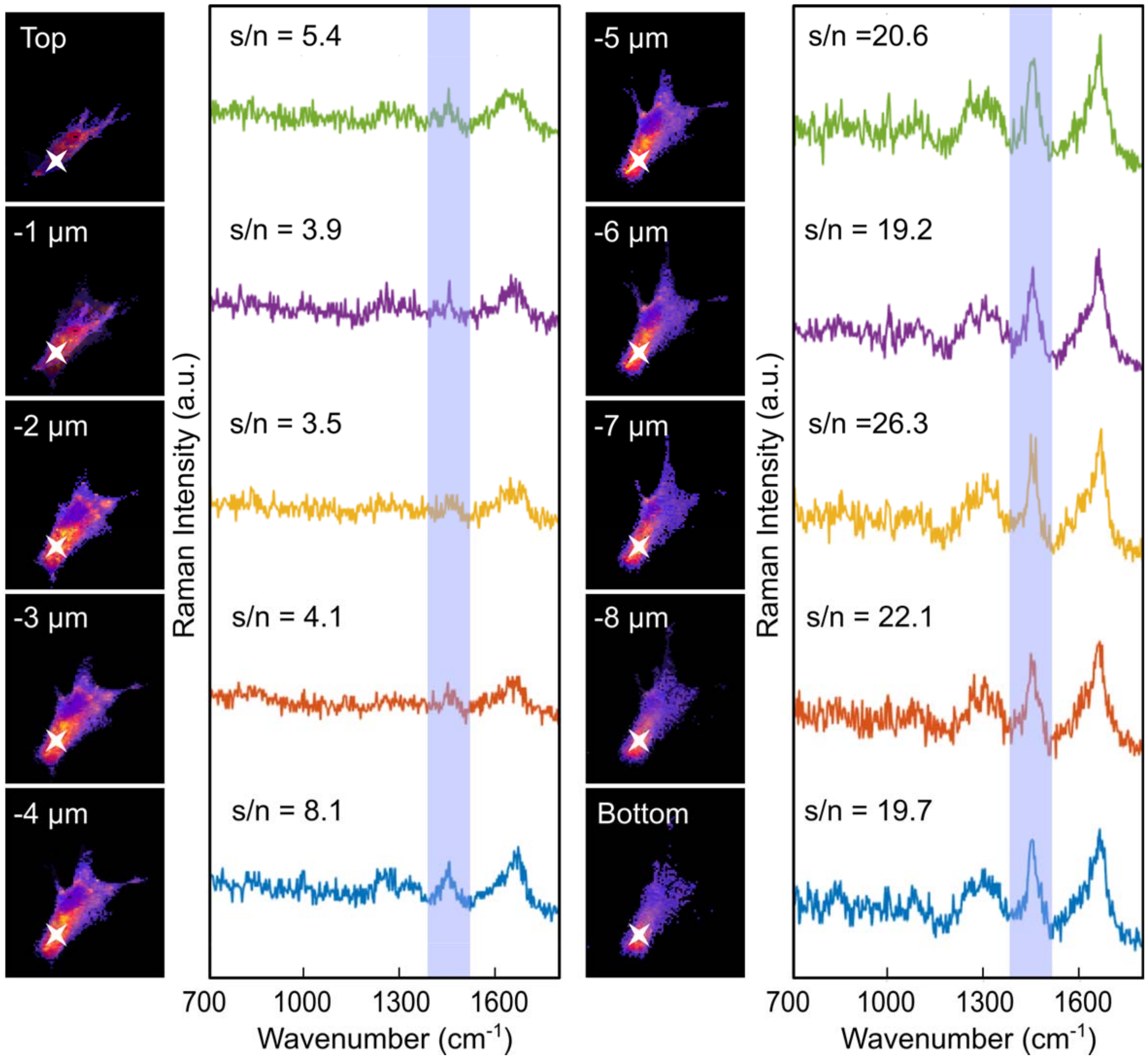

Supplementary Figure 2 | Signal-to-noise (s/n) dependence on z distance. All imaging layers from qVRI for the reconstruction of a representative MSC in a bioactive hydrogel with a 1 µm step between layers in **z**, along with representative raw Raman spectra of the same **x×y** position for all layers indicated by the white cross. S/N ratio was calculated using the 1449cm^{-1} CH_2 band (blue shaded area).

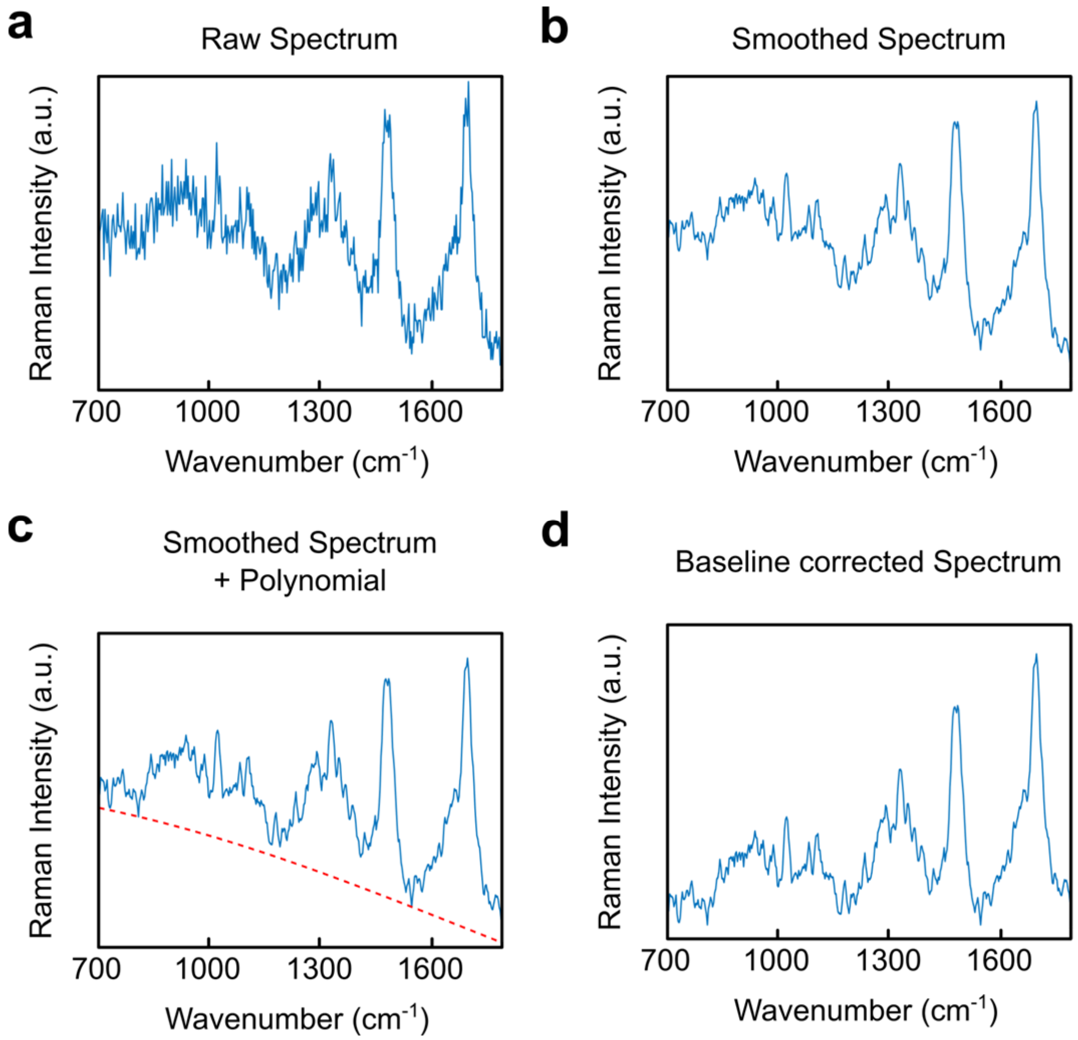

Supplementary Figure 3│ Pre-processing of representative Raman spectra. **(a)** Raw Raman spectrum **(b)** Smoothed Raman spectrum using the 2 order Savitzky–Golay algorithm with a 3 point window **(c)** Smoothed Raman spectrum with polynomial curve (dashed red) before baseline correction **(d)** Baseline corrected spectrum using a 3rd order polynomial fit.
